# A novel inhaled anesthesia technique for concurrent direct laryngoscopy in rats

**DOI:** 10.1002/ame2.70116

**Published:** 2025-12-03

**Authors:** Corinne Negvesky, Amin Mirzaaghasi, Eric M. Smith, Ashley E. Kita

**Affiliations:** ^1^ Department of Head and Neck Surgery David Geffen School of Medicine University of California Los Angeles California USA

**Keywords:** anesthesia, endotracheal intubation, laryngoscopy, rodents, vocal cords

## Abstract

The current anesthetic standard for laryngoscopy in rats utilizes injectable intraperitoneal anesthesia. Injectable anesthesia is suboptimal for short procedures due to variability in anesthesia duration and anesthetic side effects. Conversely, inhalational gas anesthesia offers precise titration with a rapid onset and offset. However, its use during laryngoscopy has not been documented due to existing administration techniques obstructing direct visualization of the larynx. The technique described here allows real‐time visualization of the rat larynx with concurrent administration of inhaled anesthetic gas. This method is particularly well‐suited for recurrent laryngeal nerve or vocal fold pathology studies, where repeat visualization of the larynx is necessary.

## Graphical abstract

Our novel nose cone anesthetic technique allows the use of gas anesthesia during concurrent direct laryngoscopy. Inhaled anesthesia not only has a better safety profile for rats, but also increases lab efficiency when compared to injectable anesthesia.

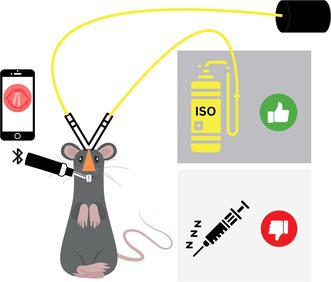

## 1 INTRODUCTION

Injectable intraperitoneal anesthesia is the current standard for anesthesia administration when sustained visualization of rat arytenoids is required, achieving excellent visualization of the vocal folds and glottis when combined with suspension microlaryngoscopy.^1^ However, it is suboptimal for short procedures due to the variability in anesthesia onset, duration, and side effects.^2^, ^3^ A literature review by Ujvary and Blebea on vocal fold injury in rat models found that ketamine and xylazine were used for maintenance anesthesia in all cases, with 82% administered intraperitoneally and 18% intramuscularly.^4^

Injectable anesthesia requires individualized dosing based on weight. The initial dose is calculated within a range, and subsequent dose adjustments are challenging. The time from initial injection of ketamine and xylazine to loss of righting reflex has been reported to vary from 1–13 min^3^, ^5^; however, we have observed cases where induction takes up to 30 min when given at recommended doses of ketamine (45–68.2 mg/kg) and xylazine (1.1–4.4 mg/kg). Furthermore, these dose ranges do not account for genetic differences, interspecies differences, or physiologic factors such as cardiac output that influence anesthetic metabolism.

Injectable anesthesia carries several risks, including an increased potential for fatal overdose due to the variable effects of initial and subsequent doses. Anesthetic depth is initially assessed and reconfirmed every 15 min throughout the procedure via loss of the toe pinch reflex, loss of corneal reflex, and decreased respiratory rate. However, studies have shown that some reflexes may persist in rats who have received injectable anesthesia.^3^ Jiron et al^5^ found the majority of rice rats anesthetized with ketamine‐xylazine were only able to obtain a surgical plane of anesthesia for 10 to 28 min of a 36‐min protocol, despite additional ketamine doses. In contrast, all rice rats anesthetized with isoflurane maintained a surgical plane of anesthesia, except for seven isolated 2‐min intervals that resolved with transiently increased isoflurane concentration.^5^ Additionally, injectable anesthesia poses more significant cardiovascular risks. Droogmans et al.^6^ reported that 80% of rats receiving intraperitoneal ketamine and xylazine developed aortic regurgitation, compared to 0% in those that received inhaled isoflurane gas.

Gas anesthesia, most commonly isoflurane in animal research, offers several advantages, including rapid onset and offset, which significantly enhances research efficiency when performing repeat short surgical procedures where laryngeal visualization is required.^2^ It is also a much safer method, as it does not rely on weight‐based dosing or redosing. Following a short preoxygenation period, anesthesia is induced and then maintained for the duration of the procedure.^2^ A longstanding challenge in studying laryngeal pathology has been the inability to use a laryngoscope with gas anesthesia due to traditional nose cone positioning. However, with the use of a specialized nose cone and an affordable digital otoscope repurposed as a rat laryngoscope, repetitive visualization of the larynx and arytenoids was achieved for procedures up to 30 min without issue.

A potential concern with isoflurane use is decreased laryngeal motion; however, we did not observe this effect with isoflurane or ketamine/xylazine anesthesia. Jackson et al^7^ found arytenoid motion was detectable for all dogs receiving acepromazine (IM), butorphanol (IM), and isoflurane mask induction anesthesia, compared to only 50% of dogs receiving ketamine (IV) and diazepam (IV) anesthesia. Another potential concern is saliva production in anesthetized rats. When bubbles of saliva accumulated over the larynx in this study, they typically cleared spontaneously within 1–2 min. Notably, Knudsen et al^8^ found that increasing isoflurane percentage in Sprague–Dawley rats resulted in reduced saliva production.

## 2 METHODS

Four Long Evans rats (two females and two males) weighing 200–250 g underwent unilateral recurrent laryngeal nerve (RLN) crush injury under ketamine/xylazine anesthesia. For the four weeks following surgery, brief repeat laryngoscopies were performed three times per week using our novel gas anesthesia method to record videos of laryngeal recovery (Table 1).

**TABLE 1:** Gender, weight, anesthetic procedures, and complications by rat.

Identifier	Gender	Weight (g)	# of ketamine/xylazine doses	# of ketamine doses	# of laryngoscopies with isoflurane	Complications
Rat 1	F	240	3	2	12	None
Rat 2	F	269	2	1	12	None
Rat 3	M	262	3	2	12	None
Rat 4	M	254	2	2	12	None

For the RLN crush surgery, rats received an intraperitoneal injection of ketamine (45–68.2 mg/kg) and xylazine (1.1–4.4 mg/kg). Once recumbency was observed, each rat was transferred to a flat surface, where a digital otoscope (Ear Wax Removal Tool, ASIN: BOBPY386YN, 4 mm diameter, Amazon, Seattle, WA), repurposed as a rat laryngoscope, was used to record arytenoid mobility prior to crush injury. A unilateral right‐sided RLN injury surgery was then performed in the same manner as described by Hernández‐Morato et al,^9^ except that jeweler's forceps were used to apply pressure for 60 s (Scientific Stainless Steel Lab Forceps with Ultrafine Tips, Labwares, Gainsville, VA). Anesthesia depth and rat oxygenation were monitored by assessing the toe pinch reflex response and vital signs at least once every 15 min, with additional ketamine injections administered as needed (approximately every 30 min). Xylazine was given with ketamine every other dose to avoid xylazine overdose. After confirming grossly reduced right laryngeal mobility after crush injury with the digital otoscope, the neck was closed. The rat was transferred to the recovery area and monitored until its breathing and activity returned to baseline (approximately 1–2 h) before being placed in a fresh cage post‐surgery.

**FIGURE 1:**
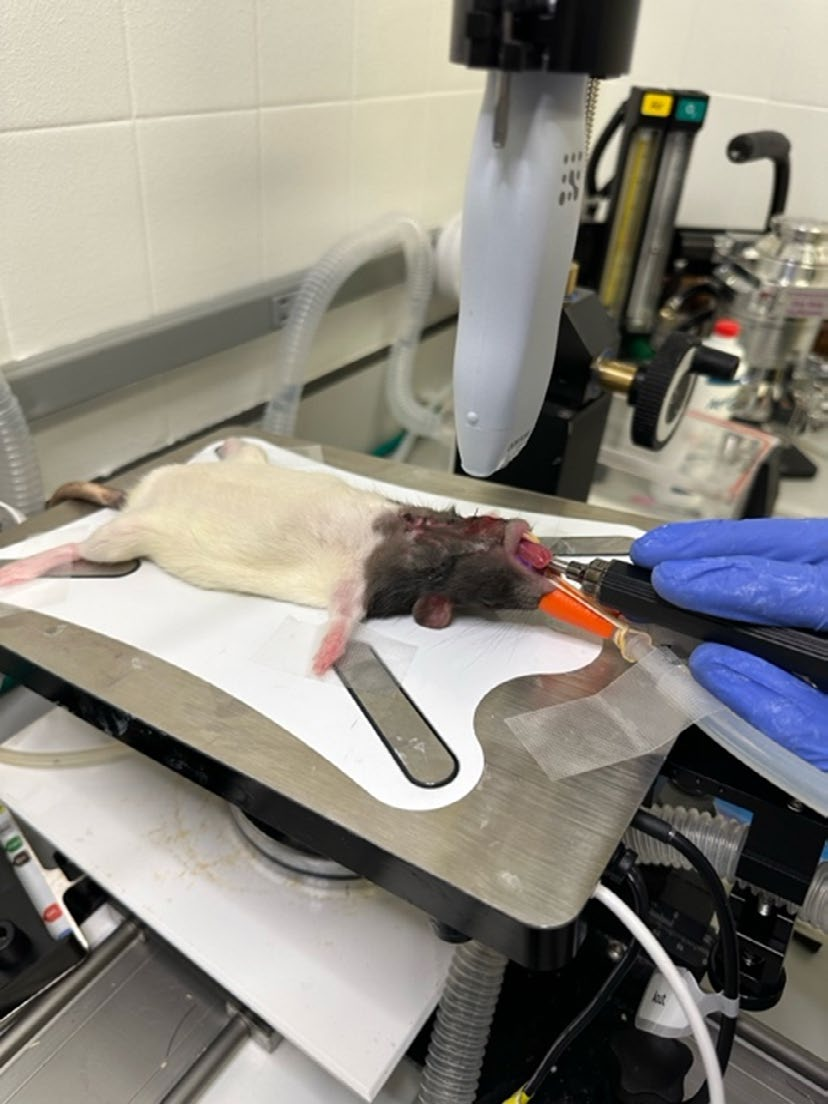
Direct laryngoscopy setup displaying the small nose cone securely fastened to the rat's nose with a rubber band connecting the upper incisor to the Y‐junction of the anesthesia tubing, allowing continuous gas anesthesia with concurrent visualization of the larynx via the digital otoscope.

For tri‐weekly laryngoscopy procedures, rats were induced with isoflurane anesthesia set to 5%. Once recumbent, each rat was transferred to a flat surface and secured in the same manner as the RLN crush surgery above. A non‐intubating nose cone (921 465, VetEquip, Marsing, ID) was affixed to the nose and secured with a rubber band (Foshine mini elastic band 1.2 inch long, 0.63 diameter, Amazon, Seattle, WA) formed into a Lark's Head knot at the Y‐junction then stretched to loop around the maxillary central incisors (Figure 1). Isoflurane anesthesia was then maintained at 2%–3.5% for the remainder of the procedure. The digital otoscope was used to visualize the larynx and track right‐sided arytenoid mobility during recovery. Once video was recorded, isoflurane was discontinued. Rats were observed to right themselves and return to normal activity levels within a few minutes. Researchers wore respirators for volatile gases including isoflurane (3M 62023) out of an abundance of caution and no acute effects of Waste Anesthetic Gas (WAG) exposure were detected. Protocols were approved by UCLA's Animal Review Committee (approval number ARC 2021‐005).

## 3 RESULTS

**FIGURE 2:**
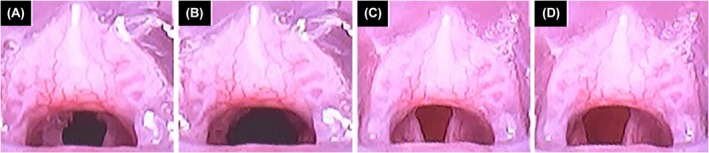
Digital otoscope view of the arytenoids in a rat under ketamine‐xylazine anesthesia prior to right‐sided recurrent laryngeal nerve (RLN) crush injury at maximal adduction (A) and abduction (B) and under isoflurane anesthesia three days post‐crush injury at maximal adduction (C) and abduction (D).

All four rats successfully underwent tri‐weekly gas anesthesia with concurrent direct laryngoscopy, completing a total of 12 short‐duration anesthetic sessions per rat (Table 1). Each session lasted between 8 and 25 min. The otoscope recording allowed for consistent visualization of the arytenoids to monitor laryngeal recovery, which was grossly observed to occur within four weeks (Figure 2). The rats remained healthy throughout the study completion and exhibited no adverse effects from the repeated gas anesthesia and laryngoscopy procedures, rapidly returning to normal behavior following each session. No anesthetic complications were observed in the animals or the staff during the inhaled anesthetic technique. While no anesthetic complications were also observed in the rats during their initial crush procedure under injectable ketamine and xylazine anesthesia, all rats required between 1 and 2 h of post‐procedure observation to resume normal activity levels, a reasonable observation period after a surgical procedure, but less ideal period of observation for repeat brief laryngoscopy procedures.

## 4 DISCUSSION

Our study successfully demonstrates a method for performing repeat laryngoscopy with direct arytenoid visualization in rats using gas anesthesia. Our non‐intubating nose cone technique provided adequate depth of anesthesia for laryngeal visualization for procedures lasting under 30 min. One limitation of our method is the necessity of prominent rat incisors to secure the nose cone with a rubber band to the Y‐connector, forming a tight seal, something which may prove difficult in rodents with less prominent incisors.

Our proposed method allows for rapid induction and recovery from anesthesia with easy intraoperative titration leading to improved laboratory efficiency for serial laryngoscopies or brief surgical procedures. Additionally, since gas anesthesia does not require weight‐based dosing or adjustments, it minimizes the risk of overdose. Variability in anesthetic session duration was attributed to the learning curve associated with the otoscope turned laryngoscope rather than issues with anesthetic depth.

One challenge we encountered was the variation in incisor size among rats. We selected larger rats as they tend to have more prominent teeth that facilitate easier fastening of the nose cone.

Given concerns for possible staff isoflurane exposure, our exploration of the duration of this method of anesthesia was limited to our institution's description of a short procedure (< 30 min). Although isoflurane is not classified as hazardous waste under Environmental Protection Agency (EPA) regulations, gas anesthesia was performed in well‐ventilated spaces with appropriate scavenging canisters and staff personal protective equipment in accordance with our institution's safe handling protocols.^10^ The research staff detected no gas leaks with the non‐intubating nose cone securely fastened to the rat's upper incisors using a rubber band. Gas anesthesia offers a fast, safe, and effective means of directly assessing laryngeal function in rats.

## AUTHOR CONTRIBUTIONS

**Corinne Negvesky:** Data curation; formal analysis; writing – original draft; writing – review and editing. **Amin Mirzaaghasi:** Conceptualization; data curation; formal analysis; investigation; methodology. **Eric M. Smith:** Conceptualization; data curation; formal analysis; investigation; methodology. **Ashley E. Kita:** Conceptualization; data curation; formal analysis; funding acquisition; investigation; methodology; project administration; writing – original draft; writing – review and editing.

## FUNDING INFORMATION

NIDCD (National Institute of Deafness and Other Communication Disorders), Grant number: K08 DC019957.

## CONFLICT OF INTEREST STATEMENT

All authors declare no potential conflict of interest.

## ETHICS APPROVAL STATEMENT

Protocols were approved by UCLA's Animal Review Committee (approval number ARC 2021‐005).

## Data Availability

Data such as anesthesia logs can be provided upon request.
